# PMMA Thin Film with Embedded Carbon Quantum Dots for Post-Fabrication Improvement of Light Harvesting in Perovskite Solar Cells

**DOI:** 10.3390/nano10020291

**Published:** 2020-02-09

**Authors:** Askar A. Maxim, Shynggys N. Sadyk, Damir Aidarkhanov, Charles Surya, Annie Ng, Yoon-Hwae Hwang, Timur Sh. Atabaev, Askhat N. Jumabekov

**Affiliations:** 1 Department of Electrical and Computer Engineering, Nazarbayev University, Nur-Sultan 010000, Kazakhstan; askar.maxim@nu.edu.kz (A.A.M.); aidarkhanov@nu.edu.kz (D.A.); annie.ng@nu.edu.kz (A.N.); 2 Department of Chemistry, Nazarbayev University, Nur-Sultan 010000, Kazakhstan; shynggys.sadyk@nu.edu.kz; 3 Nazarbayev University, Nur-Sultan 010000, Kazakhstan; charles.surya@nu.edu.kz; 4 Department of Nanoenergy Engineering and BK21 PLUS Nanoconvergence Technology Division, Pusan National University, Busan 46241, Korea; yhwang@pusan.ac.kr; 5 Department of Physics, Nazarbayev University, Nur-Sultan 010000, Kazakhstan

**Keywords:** Perovskite solar cell, PMMA, carbon quantum dots, down-conversion, light harvesting

## Abstract

Perovskite solar cells (PSCs) with a standard sandwich structure suffer from optical transmission losses due to the substrate and its active layers. Developing strategies for compensating for the losses in light harvesting is of significant importance to achieving a further enhancement in device efficiencies. In this work, the down-conversion effect of carbon quantum dots (CQDs) was employed to convert the UV fraction of the incident light into visible light. For this, thin films of poly(methyl methacrylate) with embedded carbon quantum dots (CQD@PMMA) were deposited on the illumination side of PSCs. Analysis of the device performances before and after application of CQD@PMMA photoactive functional film on PSCs revealed that the devices with the coating showed an improved photocurrent and fill factor, resulting in higher device efficiency.

## 1. Introduction

Hybrid organic–inorganic lead halide perovskites are considered to be one of the most promising materials for photovoltaic (PV) applications. In just a decade, the power conversion efficiency (PCE) of perovskite solar cells (PSCs) has improved 6-fold, reaching remarkable values of 25.2% in 2019 [1]. This is the result of tremendous efforts spent on optimization of the device fabrication process, careful tailoring of material properties used for device functional layers (perovskite photo-absorber layer, electron- and hole-transporting layers (ETL and HTL, respectively), and device structure (mesoporous, planar *p*-*i*-*n*, and planar *n*-*i*-*p*)) [2,3]. Theoretical calculations suggest that PSCs have the potential to reach efficiencies as high as ~31% [3]. Approaching the theoretical performance limit would require even further development and optimization in all aspects of device fabrication. Light management for effective utilization of incident light would be one of them.

One of the drawbacks of PSCs in terms of their light harvesting ability is the sandwich structure of the devices, which induces parasitic light absorption by the substrate (fluorine- or indium-doped tin-oxide-coated glass (glass/FTO or glass/ITO, respectively)) and other functional layers, such as ETL and HTL [4]. For instance, most of the UV light is absorbed by the ETL made of wide band gap materials, such as TiO_2_ and SnO_2_, or by the HTL (e.g., NiO_x_ in devices with an inverted structure). This, on the one hand, is beneficial for the perovskite photo-absorber layer, since it prevents degradation of the perovskite material under UV illumination. However, on the other hand, absorption of UV light by the substrate and charge-transporting layers does not allow for an effective utilization of the incident light. One way to overcome this issue without changing the structural composition of PSCs is to employ fluorescent materials with down-converting properties. These are materials that can absorb photons with higher energy (e.g., in the near-UV range) and re-emit them with lower energy (e.g., in the visible light range). Application of such functional materials to improve the light harvesting ability of the photo-absorber layer in PSCs can afford enhanced charge carrier generation and improved device performance.

**Figure 1:**
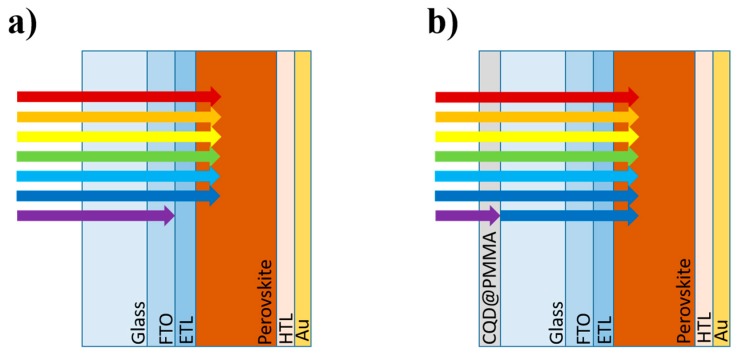
Schematic image of perovskite solar cells (PSCs) (**a**) without and (**b**) with the poly(methyl methacrylate) with embedded carbon quantum dots (CQD@PMMA) coating used for improving light harvesting in a PSC due to the down-conversion effect.

Carbon quantum dots (CQDs) with excellent fluorescent properties absorb UV light and re-emit in the visible light range. Such a unique optical property of CQDs combined with their low-cost synthesis, non-toxicity, and chemical inertness make them very attractive for electronics, sensing, and bioimaging applications [5,6,7]. For example, carbon and graphene quantum dots were utilized recently for light management in silicon-based solar cells [8,9]. In both cases, the authors reported enhancement in the performance of silicon-based solar cells, owing to the carbon/graphene quantum dots coatings that emit additional down-converted photons in the visible spectrum. A recent study reported that CQDs with a down-conversion property introduced into a mesoporous TiO_2_ film in PSCs can improve the stability (convert UV radiation to visible light) as well as the PCE of the devices [10]. Therefore, by considering the favorable down-conversion optical effects, we embedded the CQDs in a thin poly(methyl methacrylate) (PMMA) film (CQD@PMMA) and used it as an additional coating layer for PSC devices. This coating will be able to convert the UV part of the incident light to visible light, thus allowing for more light to be transmitted to the perovskite photo-absorber layer (see Figure 1).

## 2. Materials and Methods

### 2.1. Materials

High-purity materials were purchased from Alfa Aesar or Sigma-Aldrich and used as received. The FTO-coated glass substrates (~7 ohm/sq) were obtained from OPV-tech (Yingkou City, China). The organic precursors for synthesis of perovskite thin films were obtained from the GreatCell Solar.

### 2.2. Synthesis of CQDs

Phosphorus-doped CQDs were prepared according to a previously reported protocol [7]. Briefly, 0.1 g of Na_2_HPO_4_ and 1 g of dextrose were dissolved in 25 mL of deionized water. Next, the solution was placed into an Erlenmeyer flask (50 mL capacity) with a screw cap, closed, and kept at 200 °C under vigorous stirring for 1 h. The prepared black solution was passed through a 0.1 µm filter, and samples were purified and collected using a lyophilization process.

### 2.3. Preparation of CQD@PMMA Solution

As-prepared CQDs with sizes around 3–7 nm were mixed with 5 wt% PMMA in chlorobenzene (10 mg of CQDs in 10 mL of 5 wt% PMMA/chlorobenzene). The resulting mixture was sonicated for 30 min in an ultrasonic bath, and then stirred vigorously for 48 hrs at 40 °C to disperse CQDs and obtain the final brownish-colored CQD@PMMA solution.

### 2.4. Fabrication of PSCs

A glass/FTO/ETL/perovskite/HTL/gold architecture was used to fabricate PSCs. The FTO-coated glass substrates were cleaned by consecutive sonication in the detergent, deionized water (DI), and organic solvents. The substrates were then dried under a nitrogen flow and treated by UV-ozone for 30 min. The ETL was composed of a bilayer structure formed by consecutive depositions of SnO_2_ quantum dot (QD-SnO_2_) and SnO_2_ nanoparticles (NP-SnO_2_) as described below. The 0.15 M colloidal SnO_2_ quantum dot (QD-SnO_2_) solution was prepared by using SnCl_2_∙2H_2_O as the precursor dissolved in 30 mL of DI water, and left overnight under vigorous stirring in an open beaker. The prepared solution was spin coated at 3000 rpm for 30 s and thermally annealed at 150 °C for 10 min and 200 °C for 60 min. SnO_2_ nanoparticles (NP-SnO_2_) were prepared by using SnO_2_ colloidal dispersion solution. The prepared solution was spin coated at 3000 rpm for 30 s, and the samples were annealed at 150 °C for 30 min. The perovskite precursor solution was prepared by forming 1.1 M PbI_2_, 1 M formamidinium iodide (CH_5_IN_2_), 0.2 M PbBr, and 0.2 M methylammonium bromide (CH_3_NH_3_Br) in the mixture of dimethyl formamide (DMF) and dimethyl sulfoxide (DMSO) (4:1 volume ratio). Then, CsI (1.5 M stock solution) was added into the precursor solution in a volume ratio of 8:92 to form 1 mL of final perovskite precursor solution. The perovskite thin film was prepared in a nitrogen-filled glove box by a four-step process: (i) spin coating of the precursor solution at 2000 rpm for 30 s; (ii) cryogenic treatment; (iii) blow-drying under a nitrogen gas flow for 60 s; and (iv) thermal annealing at 105 °C for 30 min on a hotplate. The precursor solution for the hole transport layer (HTL) was prepared by dissolving 80 mg of 2,2’,7,7’-Tetrakis [N,N-di(4-methoxyphenyl)amino]-9,9’-spirobifluorene (Spiro-OMeTAD), 954 μL of chlorobenzene, 29 μL of 4-tert-Butylpyridine, and 17.5 μL of Bis(trifluoromethane)sulfonimide lithium salt in acetonitrile with a concentration of 520 mg/mL. The prepared solution was spin coated at 3500 rpm for 30 s. Gold electrodes (70 nm) were thermally evaporated through a shadow mask at a vacuum pressure of 10^−7^ Torr to complete fabrication of PSCs (Glass/FTO/SnO_2_/Cs_0.05_(MA_0.17_FA_0.83_)_0.95_Pb(I_0.84_Br_0.16_)_3_/spiroMeOTAD/Au) [11].

### 2.5. Deposition of CQD@PMMA Coating on PSCs

In order to deposit CQD@PMMA coating on PSCs, freshly prepared CQD@PMMA solution was spin coated (30 s under various spinning speed conditions) on the glass slide of already fabricated PSC devices at room temperature. For each deposition speed, at least 15 devices were tested. The estimated number of CQD particles per 1 × 1 cm^2^ geometric area is ~1.48 × 10^12^.

### 2.6. Characterization

A transmission electron microscope (TEM) (JEM 2010F, JEOL Ltd., Tokyo, Japan) was used for morphology and size analysis of CQDs. Fluorescent measurements were performed using a Cary Eclipse Fluorescence Spectrophotometer (Agilent Technologies Inc., Santa Clara, CA, USA). Transmittance measurements were performed using a Lambda 1050 spectrophotometer (Perkin Elmer Inc., Waltham, MA, USA) equipped with an integrating sphere. The *J–V* characteristics of the devices were measured by using a Keithley 2400 sourcemeter (Tektronix Inc., Beaverrton, OR, USA) under an AAA class ORIEL Sol3A solar simulator (Newport Corporation, Irvine, CA, USA) equipped with an Air Mass (AM) 1.5 filter at 100 mW/cm^2^. The light intensity was calibrated by a Si reference cell from Newport (Newport Corporation, Irvine, CA, USA).

## 3. Results and Discussion

**Figure 2:**
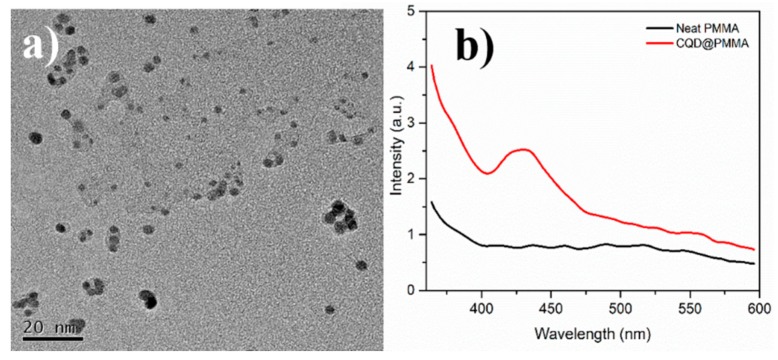
(**a**) TEM image of prepared carbon quantum dots (CQDs), and (**b**) fluorescence emission of neat poly(methyl methacrylate) (PMMA) and CQD@PMMA coatings deposited on thin microscope glass slides.

The TEM analysis of CQD samples confirmed a narrow size distribution in the range of 3–7 nm (Figure 2a). The down-converting property of the CQD@PMMA films was tested using fluorescence spectroscopy. For this, freshly made CQD@PMMA solution was spin coated on thin microscope glass slides and fluorescence emission spectra of the samples were recorded. The emission spectra of the coatings were tested at the excitation wavelength of 320 nm. As shown in Figure 2b, the emission spectra of a CQD@PMMA film shows a distinct peak centered at 430–440 nm, which is attributed to a typical emission range of CQDs [7]. The emission spectra of a neat PMMA film deposited on a thin microscope glass slide were also recorded for comparison. The absence of an emission peak in the neat PMMA film indicates that the emission peak in the CQD@PMMA film originates from the CQDs embedded in the PMMA matrix.

**Figure 3:**
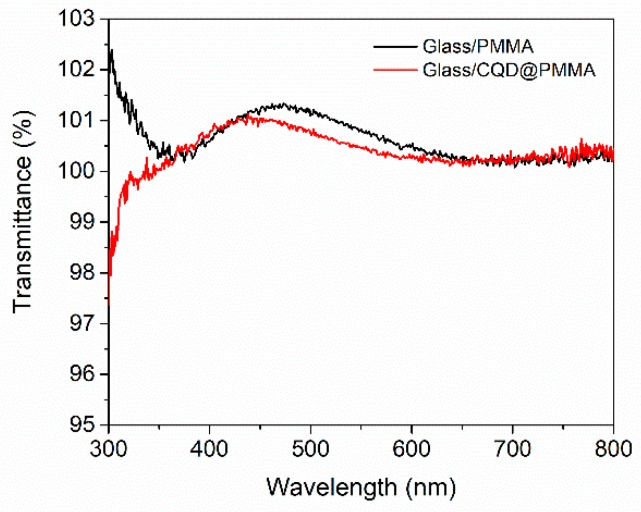
Transmittance of neat PMMA and CQD@PMMA coatings on thin microscope glass slides.

Figure 3 shows the transmittance spectrum of neat PMMA and CQD@PMMA films deposited on thin microscope glass slides for the measurement range of 300–800 nm. The CQD@PMMA film has lower transmittance in the near-UV region (<350 nm) compared to the neat PMMA film, which suggests absorption of UV light by the CQDs embedded in the PMMA matrix.

**Figure 4:**
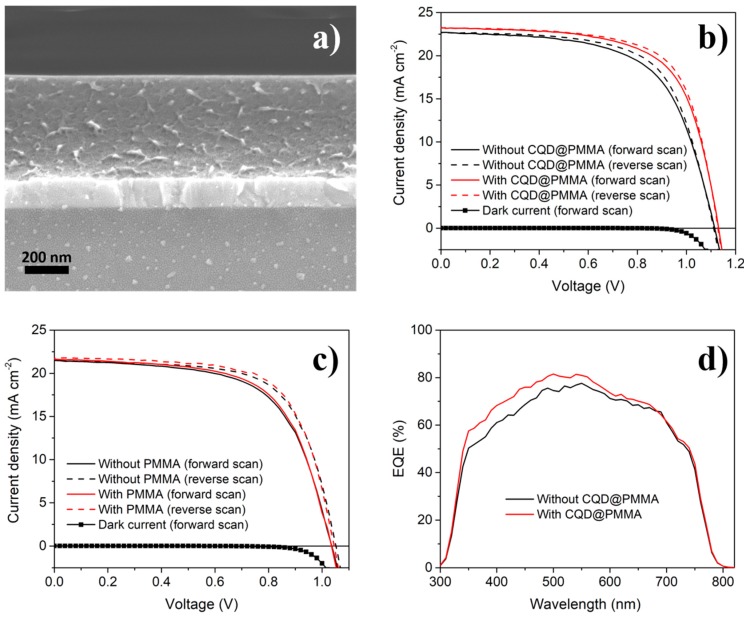
(**a**) Cross-sectional SEM image of a CQD@PMMA coating deposited onto a fluorine-doped tin oxide (FTO) substrate at 3000 rpm. (**b**) *J–V* characteristics (scan rate 0.5 V s^−1^) of a PSC device before and after application of a CQD@PMMA coating tested under 1 sun illumination and in the dark (blue solid line). (**c**) *J–V* curves of a PSC device before and after application of a neat PMMA layer. (**d**) External quantum efficiency (EQE) spectra of a PSC before and after application of a CQD@PMMA layer.

In the next stage, CQD@PMMA coatings were applied to PSCs in order to evaluate their ability to enhance the light harvesting efficiency of the devices. For this, a PMMA/chlorobenzene mixture with dispersed CQDs was spin-coated (30 s at 3000 rpm) on the illumination side of prefabricated PSCs. The thickness of the CQD@PMMA coating was around 480 nm (Figure 4a). To evaluate the effect of CQD@PMMA coating on device performance, *J–V* characteristics of PSCs before and after their application were measured. Figure 4b shows a comparison of *J–V* curves, both forward and reverse, for devices before and after application of a CQD@PMMA coating. The active area of the device was 0.075 cm^2^. The *J–V* curves show that the device with the CQD@PMMA coating resulted in a higher photocurrent (*J_SC_*) compared to the device without the CQD@PMMA coating. The relative increase for *J_SC_*, which was around 3%, was consistent with the transmittance measurements presented in Figure 3. The *J–V* curves also show that there was a notable enhancement (around a 6% relative increase) in fill factor (*FF*), and some minor improvements (around a 1% relative increase) in open-circuit voltage (*V_OC_*) after the application of the CQD@PMMA layer on the device (see Table 1).

Control experiments were also performed, in which the effect of neat PMMA coating on the light harvesting ability of PSCs was investigated. For this, we obtained the *J–V* characteristics of PSCs before and after the application of a neat PMMA coating (deposited via spin-coating for 30 s at 3000 rpm). This was done in order to separate and estimate the effect of the PMMA layer alone on the performance of the devices. Comparison of the *J–V* curves for a device before and after application of the neat PMMA coating shows that the PMMA coating alone had a negligible or no effect on the performance of the device (Figure 4c). This indicates that the device performance enhancement observed in the case of CQD@PMMA coating indeed originates from the CQDs embedded in the PMMA matrix—i.e., due to the down-conversion effect of CQDs, effectively converting UV to visible light, affording more photons to pass into the perovskite photo-absorber layer, and, thus, generating more charge carriers.

The external quantum efficiency (EQE) measurements were also performed on PSCs to obtain their optical spectral response before and after application of CQD@PMMA coating. Figure 4c shows EQE spectra of a device measured before and after application of a CQD@PMMA coating. The comparison of EQE spectra indicates that after deposition of the CQD@PMMA coating, the EQE response of the device in the blue region has improved [8,9,10]. The calculated *J_SC_* from EQE spectra obtained before and after deposition of the CQD@PMMA coating results in values of 16.31 mA cm^−2^ and 17.14 mA cm^−2^, respectively, which exhibits the consistent improvement in *J_SC_* for the device after deposition of the CQD@PMMA coating. It is noteworthy that an ~26%–28% discrepancy was observed between the calculated *J_SC_* from the EQE spectra and the measured *J_SC_* from the solar simulator. The underestimation of EQE commonly happens in PSCs. This can be due to the barrier for the photocurrent of PSC becoming dominant under the monochromatic illumination with low light intensity during the EQE measurement, while the barrier will be alleviated by photodoping of the perovskite under the strong illumination. Nevertheless, the enchantment trend, as determined from EQE, is consistent with the trend of the *J–V* characteristics measured under 1 sun illumination.

Finally, we investigated the dependence of the PSC performance on the thickness of the CQD@PMMA coating. For this, we deposited a CQD@PMMA coating on the glass side of PSCs at various spinning speeds. The performance of the devices was measured before and after application of the CQD@PMMA coating, and relative changes in *J_SC_*, *FF*, and *V_OC_* and the efficiency of the devices were investigated. The relative change for photovoltaic parameters is given as a percentage; i.e., it indicates to what extent a certain photovoltaic parameter is changed (improved or worsened) after the deposition of CQD@PMMA coating.

**Figure 5:**
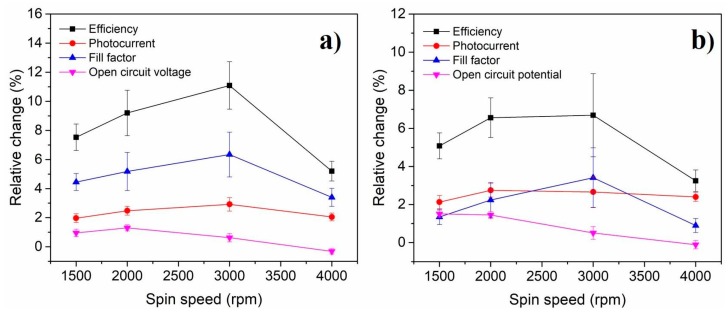
Dependence of relative change in the photovoltaic parameters on the spinning speed used to deposit a CQD@PMMA coating on PSCs. Key photovoltaic parameters extracted from *J–V* curves measured in (**a**) forward and (**b**) reverse scan directions.

Figure 5 shows the dependence of relative change in the photovoltaic parameters on the spinning speed used to deposit CQD@PMMA coating on PSCs. In order to verify the reproducibility of the PSCs, at least 15 devices for each spinning speed condition were examined. Similar ranges of deviations in photovoltaic parameters of the devices were observed among all spinning conditions, which were predicted due to the high sensitivity of the device performance to the fabrication ambient. Nevertheless, the trend of the device performance with varying thicknesses of the CQD@PMMA coating can clearly be observed. Figure 5a,b show the device performance parameters extracted from *J–V* curves measured in forward and reverse scan directions, respectively. The curves for device efficiency for forward and reverse scan directions show that there is a gradual increase from a lower spinning speed up to 3000 rpm, and then it drops. This trend, especially for forward scan directions (see Figure 5a), originates from a similar behavior in relative changes of *J_SC_* and *FF*. The initial increase up to 3000 rpm and the following decrease in the relative change of *J_SC_* in Figure 5a,b can be attributed to the thickness of the CQD@PMMA coating. Thicker CQD@PMMA coating has many CQD particles, which leads to transmission losses, while a CQD@PMMA coating that is too thin is not effective in converting UV into visible light. Based on this assumption, we can cautiously suggest that 3000 rpm for deposition of CQD@PMMA coating, yielding a thickness of around 480 nm, is the optimal value for spinning speed. Meanwhile, a similar dependence of *FF* on spinning speed—i.e., an initial increase up to 3000 rpm and a subsequent decrease at a higher spinning speed—for both scan directions is not as intuitive to explain. However, a similar trend was reported by Jin et al. [10] in related studies. A possible explanation for this phenomenon could be the UV-filtering effect of CQDs, which prevents the perovskite layer from photodegradation. However, a more detailed investigation of this phenomenon is required and will be the subject of future studies. The relative changes in *V_OC_* for both scanning directions, however, show very subtle improvements after deposition of CQD@PMMA coating, and slowly decrease with a higher spinning speed, indicating a negligible relative change in *V_OC_* for thinner CQD@PMMA layers.

## 4. Conclusions

In conclusion, we developed an easy-to-handle, low-cost, and solution-processable method to fabricate photoactive functional coating for sandwich-structured PSCs to enhance their light harvesting ability and improve device performance. The photoactive functional coating consists of a PMMA matrix embedded with CQDs that possess down-conversion fluorescent properties. The application of this coating on PSC devices showed an improved photocurrent and fill factor, resulting in an overall performance increase. The method is simple and straightforward to apply and can afford further fine-tuning and/or modification to fabricate functional thin films with the desired properties and morphology. In addition, this low-cost and solution-based method offers ease of scale-up of fabrication, which makes it very attractive for future industrial manufacturing.

## Figures and Tables

**Table 1:** Photovoltaic parameters for a PSC device before and after application of a CQD@PMMA layer tested under 1 sun illumination.

CQD@PMMA Layer	Scan Direction	*J_SC_* (mA cm^−1^)	*V_OC_* (V)	*FF* (%)	Efficiency (%)
Without	forward	22.69	1.11	61.98	15.67
Without	reverse	22.63	1.11	65.29	16.4
With	forward	23.25	1.13	65.76	17.29
With	reverse	23.21	1.13	68.19	17.86
